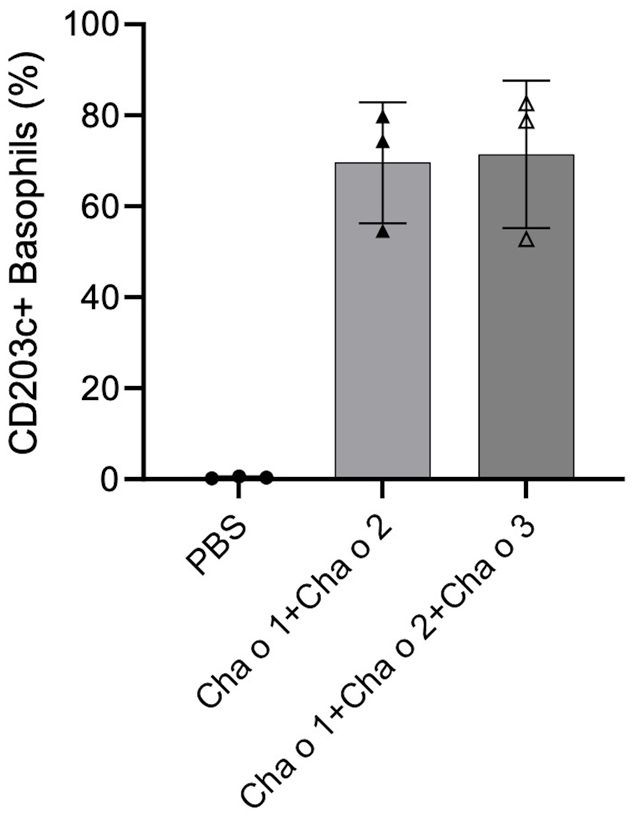# Cha o 3, a cypress pollen allergen, does not activate basophils in Japanese cypress pollinosis

**DOI:** 10.1016/j.jacig.2023.100198

**Published:** 2023-12-01

**Authors:** Yoshiaki Kobayashi, Keisuke Suzuki, Minoru Tateno, Yuki Nakamura, Kayoko Ishimaru, Yuka Nagasaka, Daiju Sakurai, Katsuyo Ohashi-Doi, Atsuhito Nakao

**Affiliations:** a Department of Immunology, Faculty of Medicine, University of Yamanashi, Yamanashi, Japan; c Department of Otorhinolaryngology, Head and Neck Surgery, University of Yamanashi, Yamanashi, Japan; d Yamanashi GLIA Center, University of Yamanashi, Yamanashi, Japan; b Torii Pharmaceutical Co, Ltd, Tokyo, Japan; e Atopy Research Center, Juntendo University School of Medicine, Tokyo, Japan

**Keywords:** Japanese cypress pollinosis, Cha o 3, basophil activation test

## Abstract

**Background:**

In Japan, pollinosis caused by the Japanese cypress (JCy) *Chamaecyparis obtusa* is among the very common seasonal allergies. In JCy pollinosis, Cha o 1 is the first major allergen, and Cha o 2 is the second major allergen. Recently, Cha o 3 was identified as a new JCy pollen allergen in JCy pollinosis. However, the relative contribution of Cha o 3 to JCy pollinosis compared with that of Cha o 1 and that of Cha o 2 has not been fully elucidated.

**Objective:**

This study aimed to clarify the allergenicity of Cha o 3 compared with that of Cha o 1 and Cha o 2 in JCy pollinosis.

**Methods:**

We recruited 27 patients with JCy pollinosis and performed the basophil activation test (BAT) with native (n) Cha o 1, Cha o 2, and Cha o 3 purified from JCy pollen. In addition, we a performed JCy-specific IgE suppression test.

**Results:**

In the BAT, 26 of 27 patients (96%) and 18 of 27 patients (67%) showed positive basophil activation in response to n Cha o 1 and n Cha o 2, respectively, as judged by CD203c expression. Little CD203c expression in response to n Cha o 3 was seen. The presence of n Cha o 3 marginally reduced the titer levels of JCy-specific IgE.

**Conclusion:**

Cha o 3 showed little ability to activate basophils and suppress JCy-specific IgE titers compared with Cha o 1 or Cha o 2 in patients with JCy pollinosis. Thus, Cha o 3 may not be a major allergen in JCy pollinosis.

## Introduction

Pollens from species of the Cupressaceae family are among the most important causes of respiratory allergies worldwide.^1^ In North America, Mountain cedar (*Juniperius ashei*) is a major source of pollen of the genus *Cupressus* that causes allergic reactions,^2^ whereas in the Mediterranean region, Arizona cypress (*Cupressus arizonica*) and Mediterranean cypress (*Cupressus sempervirens*) release pollens that cause allergic reactions.^3^

In Japan, pollinosis caused by the Japanese cypress (JCy), which belongs to the Cupressaceae family, is among the very common seasonal allergies.^4^^,^^5^ Around 15% of Japanese people experience symptoms of allergic rhinitis and conjunctivitis when JCy pollen is dispersed in Japan from March through early May. In JCy pollinosis, *Chamaecyparis obtusa* (Cha o) 1 is the first major allergen and Cha o 2 is the second major allergen.^4^^,^^5^ Cha o 1 is a glycoprotein comprising 374 amino acids; it is observed with molecular weights of 45 and 50 kDa by SDS-PAGE, which likely reflects glycosylation difference. Cha o 2 is expressed as a 514–amino acid proprotein, including a 50-mer signal peptide; mature Cha o 2 runs as an approximately 42- to 45-kDa band on SDS-PAGE. Cha o 1 and Cha o 2 are presumed to have pectate lyase activity or polygalacturonase activity based on their amino acid homology to pectate lyase allergens or polygalacturonases of other Cuppressaceae pollens from around the world, respectively.

In 2016, Cha o 3, which has little homology to Cha o 1 and Cha o 2 in terms of amino acid sequences, was identified as a new JCy pollen allergen in patients with JCy pollinosis.^6^ Interestingly, Cha o 3 contains a cellulase domain and has been suggested to be the first plant cellulase with allergenic activity. In the first report on Cha o 3, 87.5% of patients with JCy pollinosis were positive for Cha o 3–specific IgE, and 3 of 3 patients with JCy pollinosis showed a level of basophil activation in response to Cha o 3 that was equal to or even higher than that in response to Cha o 1.^6^ However, there has not been enough evidence regarding the relative contribution of Cha o 3 to JCy pollinosis compared with that of Cha o 1 and Cha o 2.

This study aimed to clarify the allergenicity of Cha o 3 compared with that of Cha o 1 and Cha o 2 in JCy pollinosis. For this purpose, we recruited 27 patients with JCy pollinosis and performed a basophil activation test (BAT) with native (n) Cha o 1, n Cha o 2, and n Cha o 3 purified from JCy pollen. In addition, we performed a JCy-specific IgE suppression test using serum samples from the 27 patients. Details on our methods are available in this article's Online Repository at www.jaci-global.org.

## Results and discussion

**Supplementary Figure E1:**
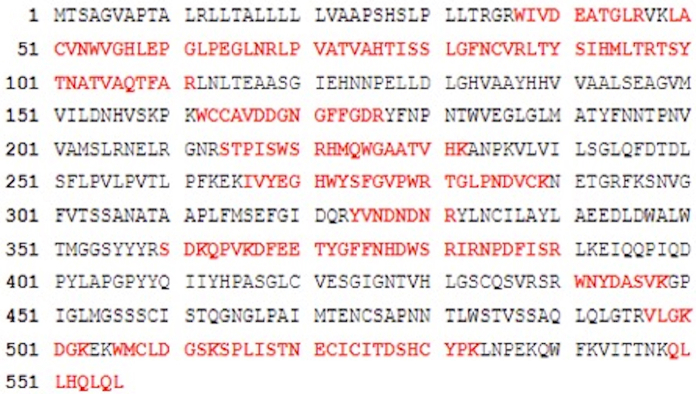


**Supplementary Figure E2:**
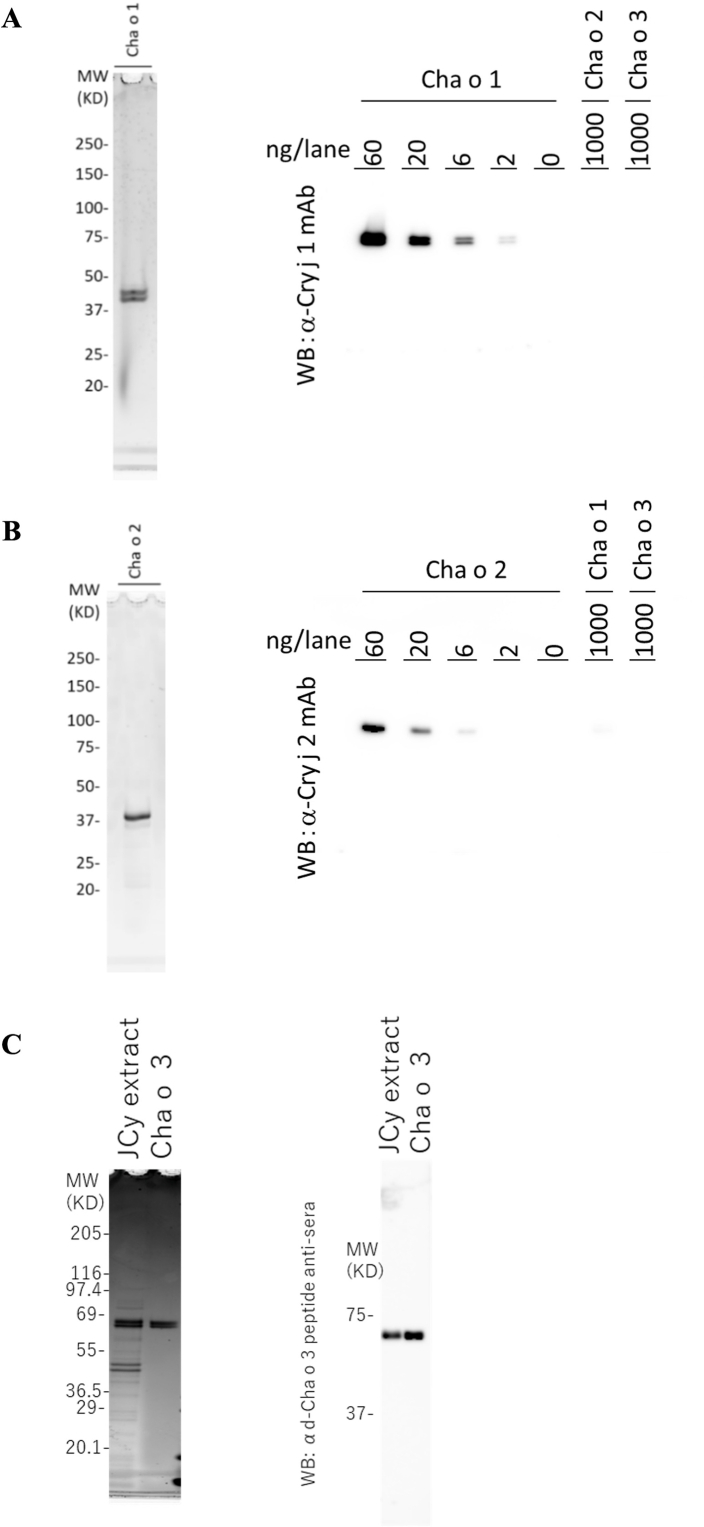


**Supplementary Figure E3:**
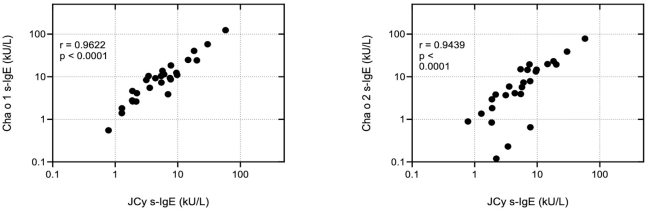


We first validated the identify of n Cha o 3 by liquid chromatography–tandem mass spectrometry (see Fig E1 in this article's Online Repository at www.jaci-global.org) and confirmed the specificity of n Cha o 1, n Cha o 2, and n Cha o 3 by Western blot analysis with anti–*Cryptomeria japonica* (Cry j) 1 (the first major allergen of Japanese cedar [JC] pollen, which shared allergenicity with Cha o 1),^5^ anti–Cry j 2 (the second major allergen of JC pollen, which shared allergenicity with Cha o 2),^5^ and anti–Cha o 3 antibody, respectively (see Fig E2 in this article's Online Repository at www.jaci-global.org). ImmunoCAP assay using n Cha o 1 and n Cha o 2 revealed that of the 27 patients, 26 (96 %) had n Cha o 1–specific IgE and 24 (89%) had n Cha o 2–specific IgE (Table I). In addition, the serum titers of n Cha o 1– or n Cha o 2–specific IgE were correlated with the serum JCy-specific IgE titers, respectively (see Fig E3 in this article's Online Repository at www.jaci-global.org).

**Table I:** Patient profiles

Characteristic	Patients with JCy pollinosis
	(N=27)
Male, no. (%)	18 (67)
Age (y), mean ± SD	24.6 ± 5.1
Duration of JCy pollinosis (y), mean ± SD	13.3 ± 6.1
Specific IgE level (kU/L), JCy extract	
≥0.7, no. (%)∗	27 (100)
Mean ± SD	8.9 ± 11.6
Specific IgE level (kU/L), n Cha o 1	
≥0.7, no. (%)∗	26 (96)
Mean ± SD	16.0 ± 24.5
Specific IgE level (kU/L), n Cha o 2	
≥0.7, no. (%)∗	24 (89)
Mean ± SD	11.4 ± 15.9

∗ Specific IgE value was measured by ImmunoCAP assay. JCy pollen sensitization was defined as each specific IgE level of 0.7 kU/L or higher.

**Fig 1:**
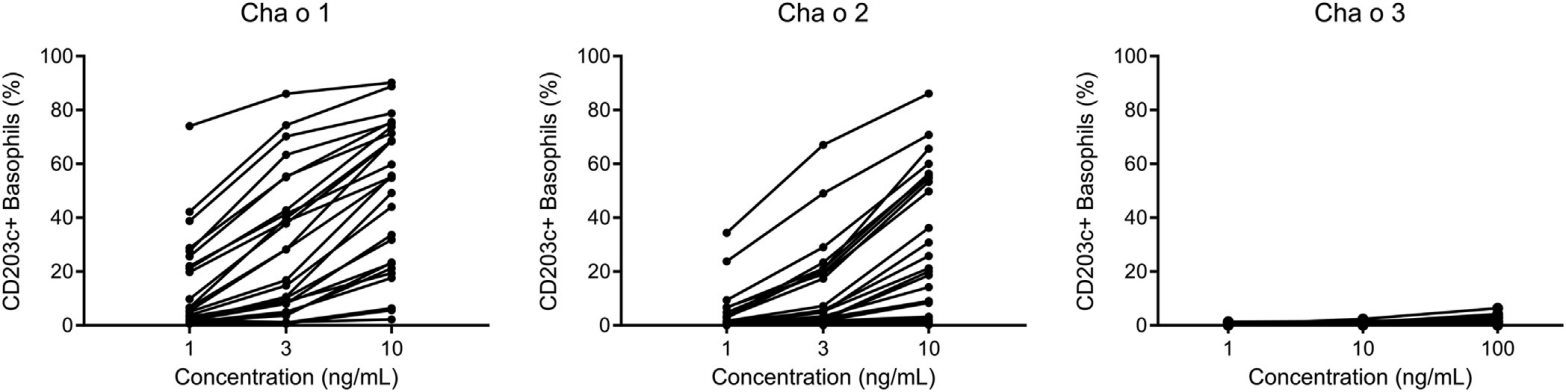
BATs with n Cha o 1, n Cha o 2, and n Cha o 3. The BAT was performed at concentrations of 1, 3, and10 ng/mL for n Cha o 1 and n Cha o 2 and 1, 10, and 100 ng/mL for n Cha o 3.

**Supplementary Figure E4:**
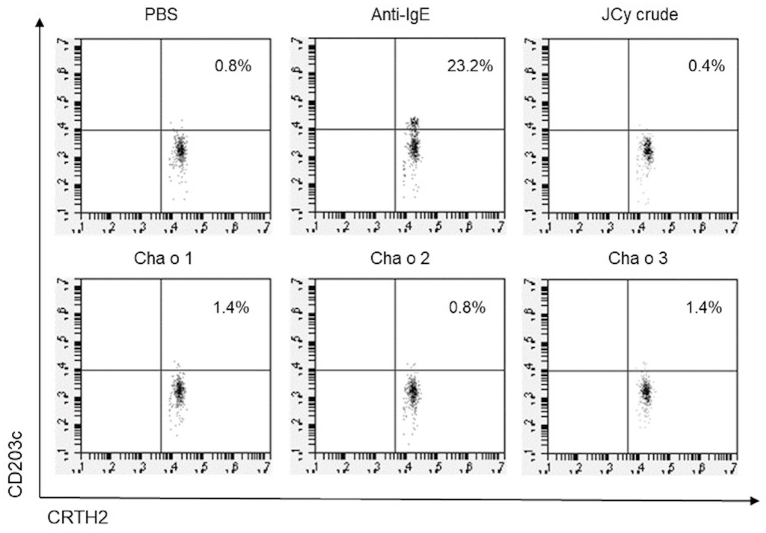


In the BAT, 26 of 27 patients (96%) showed positive basophil activation in response to 10 ng/mL of n Cha o 1 and 18 of 27 patients (67%) showed positive basophil activation in response to 10 ng/mL of n Cha o 2 (a ≥5% increase of CD203c^+^ basophils compared with PBS following the stimulations was considered positive) (Fig 1**)**. However, little or no detectable CD203c expression in response to n Cha o 3 was seen even after stimulation with a 10-fold higher concentration (100 ng/mL) than used for the allergens n Cha o 1 and n Cha o 2 (Fig 1). As a negative control, we confirmed that basophils derived from a normal subject did not respond to n Cha o 1, n Cha o 2, or n Cha o 3 (see Fig E4 in this article's Online Repository at www.jaci-global.org). Furthermore, the addition of n Cha o 3 did not affect n Cha o 1– or n Cha o 2–induced basophil activation in 3 patients with JCy pollinosis (see Fig E5 in this article's Online Repository at www.jaci-global.org), suggesting that n Cha o 3 did not contain any contaminants affecting basophil activation.

**Fig 2:**
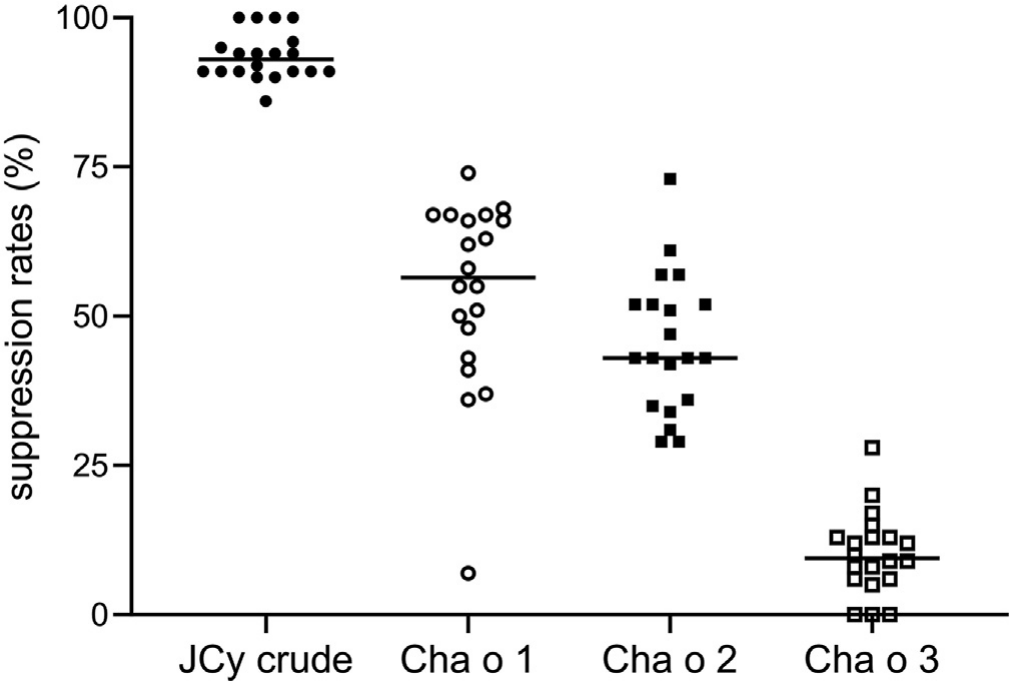
JCy-specific IgE inhibition test performed with n Cha o 1, n Cha o 2, and n Cha o 3. The inhibition assay was assessed by Japanese cypress ImmunoCAP assay in the presence of PBS, JCy extract, n Cha o 1, n Cha o 2, or n Cha o 3.

We also performed a JCy-specific IgE suppression test using the serum samples from the 27 patients (Fig 2). The level of suppression was assessed by JCy ImmunoCAP assay in the presence of competing allergen (JCy crude extract, n Cha o 1, n Cha o 2, and n Cha o 3) or PBS in the liquid phase. The suppression rates were calculated by the following formula: 1 – each allergen-containing titer/PBS-containing titer. The titers according to the JCy ImmunoCAP assay were as follows: 13.7 ± 17.9 kU/L in the presence of PBS, 0.9 ± 1.1 kU/L in the presence of JCy crude extract (94% suppression), 5.7 ± 5.8 kU/L in the presence of n Cha o 1 (54% suppression), 7.6 ± 10.5 kU/L in the presence of Cha o 2 (46% suppression), and 12.3 ± 16.1 kU/L in the presence of Cha o 3 (10% suppression). The results were consistent with the BAT results.

The current findings of the BAT and the JCy-specific IgE suppression test are in striking contrast to the findings of previous reports.^6^, ^7^, ^8^ In their original study, Osada et al showed that 87.5% of patients with JCy pollinosis were positive for Cha o 3–specific IgE and 3 of 3 patients with JCy pollinosis showed positive basophil activation in response to Cha o 3 to the same or even higher extent than to Cha o 1.^6^ In their subsequent studies, 13 of 14 (92.9%) and 18 of 18 (100%) patients with JCy pollinosis were BAT-positive to Cha o 3.^7^^,^^8^ In addition, PBMCs from patients with JCy pollinosis produced significant IL-5 after Cha o 3 stimulation.^8^ Thus, on the basis of the findings of these studies, Cha o 3 appears to be a major allergen in JCy pollinosis.

The reason(s) for the difference between the previous studies and the current study remains unclear. We assume several possibilities, including patient cohort and purity of allergens. As for patient cohort, this study recruited patients in Yamanashi Prefecture, which is a highland prefecture to the west of Tokyo, whereas the previous study recruited patients who live in Saitama Prefecture, which is an inland prefecture located to the north of Tokyo.^6^ There are regional differences in the prevalence of sensitization to JCy in Japan, and Yamanashi Prefecture is the region with the highest prevalence of positivity for JCy.^9^ Therefore, the patient cohort might not be the primary factor responsible for the different outcomes between the 2 studies. As for the purity of allergens, we validated the identity of n Cha o 3 by liquid chromatography–tandem mass spectrometry as well as by using rabbit polyclonal antibody against Cha o 3 (see Figs E1 and E2). We also excluded contamination of n Cha o 3 by Western blot analysis showing that neither anti–Cry j 1 antibody nor anti–Cry j 2 antibody reacted to even a high dose (1000 ng/lane) of n Cha o 3 (see Fig E2) as well as by BAT analysis showing that basophils from a normal subject did not respond to n Cha o 3 (see Fig E4) and the addition of n Cha o 3 did not affect n Cha o 1– or n Cha o 2–induced basophil activation (see Fig E5). This issue should thus be clarified in future studies.

Recently, Yonekura et al reported that allergic symptoms caused by JCy pollen were suppressed by JC pollen sublingual immunotherapy tablets containing the major allergens Cry j 1 and Cry j 2, which have about 80% amino acid sequence homology to Cha o 1 and Cha o 2, respectively.^10^ The JC pollen sublingual immunotherapy tablets contain little Cry j 4 JC allergen having amino acid sequence homology to Cha o 3.^4^ The clinical findings may be consistent with the current findings that most of the patients with JCy pollinosis did not show responses to Cha o 3.

In summary, this study suggests that Cha o 3 may not play a major role in JCy pollinosis. Further extensive studies are thus required to establish Cha o 3 as an important allergen in JCy pollinosis and to advance our understanding of JCy pollinosis and its allergen immunotherapy.

## Disclosure statement

Supported by the 10.13039/501100001700Ministry of Education, Culture, Sports, Science and Technology, Japan (grant-in-aid for scientific research 22K19427 [to A.N.]) and Torii Pharmaceutical Co, Ltd (to A.N.).

Disclosure of potential conflict of interest: A. Nakao received a research grant from Torii Pharmaceutical Co, Ltd. The rest of the authors declare that they have no relevant conflicts of interest.

## Figures and Tables

**Supplementary Figure E5:**