# Supplementary material for: Cha o 3, a cypress pollen allergen, does not activate basophils in Japanese cypress pollinosis
Source: J Allergy Clin Immunol Glob. 2023 Dec 1;3(1):100198. doi: 10.1016/j.jacig.2023.100198 (PMC10753085; doi:10.1016/j.jacig.2023.100198)
Supplement: Supplementary data [file mmc1.docx]

**Online Repository (Methods)**

**Patients**

We recruited twenty-seven Japanese cypress (JCy) pollinosis patients and one normal subject at the University of Yamanashi Hospital with written informed consent following the Helsinki Declaration of the World Medical Association. JCy pollinosis are defined by the presence of seasonal allergic symptoms and the serum JCy-specific IgE titer of ≥0.7kU/L (ImmunoCAP, Phadia 200, Thermo Fischer Scientific). We regarded patients defined as Class 2-6 in the class system based on the ImmunoCAP (Class 0: from 0 to < 0.35 kU_A_/L; Class 1: from 0.35 to < 0.7 kU_A_/L; Class 2: from 0.70 to < 3.5 kU_A_/L; Class 3: from 3.50 to < 17.5 kU_A_/L; Class 4: from 17.5 to < 50 kU_A_/L; Class 5: from 50 to < 100 kU_A_/L; and Class 6: from ≥ 100 kU_A_/L) as those who were sensitized to JCy pollen.^E1^ The patient profile is described in **Table 1**. None of the patients were treated with any medication at least 1 month before and during this study. The ethics committee of the University of Yamanashi Faculty of Medicine approved this study (No. 2504).

**Preparation of Cha o allergens**

*Purification of Cha o 1 (native [n] Cha o 1)*

Chamaecyparis obtusa pollen (Yamizo pollen study group, Ibaraki, Japan) was extracted at 1:20 (w/v) in 40 mM NH4HCO3, pH 7.8, for 3 hours at 4°C with gentle stirring. Supernatant was obtained by centrifugation (20,000xg, 30 min, 4C). Proteins were precipitated in the presence of 80% (NH4)2SO4. The precipitate was dissolved in small amount of 50 mM Tris-Cl (pH 7.8), dialyzed against 1.5 M (NH4)2SO4, 50 mM Tris-Cl (pH 7.8) and applied onto a Butyl-S column (Cytiva, Marlborough, MA, US). Flow-through fraction was applied onto a HiTrap Phenyl HP column (Cytiva). Proteins were eluted from the column with a gradient of decreasing concentration of (NH4)2SO4. Pooled fraction was dialysed against phosphate buffered saline (PBS). Concentrated protein (Vivaspin 15R (MWCO: 10,000HY, Sartorius) was further separated by a HiLoad 26/600 Superdex 75 pg gel filtration column (Cytiva). Fractions were examined by Western blotting with anti-Cry j 1 monoclonal antibody (Torii pharmaceutical) to choose fractions including Cha o 1 for further purification. Protein concentration was determined by the BCA method. Images were analyzed with ImageQuant TL (Cytiva) to estimate the purity of the protein.

*Purification of Cha o 2 (n Cha o 2)*

Cha o 2 was purified from Chamaecyparis obtusa pollen (Yamizo pollen study group) according to the previously described method with modification. ^E2-4^ Pollen was extracted at 1:20 (w/v) in 40 mM NH4HCO3, pH 7.8, for 3 hours at 4°C with gentle stirring. The precipitate obtained by centrifugation (20,000xg, 30 min) at 4°C was further extracted at 1:20 (w/v) in 40 mM NH4HCO3, pH 7.8, 0.5 M NaCl for 24 h at 4°C with vigorous stirring. Supernatant was obtained by centrifugation (20,000xg, 30 min) at 4°C. Protein was precipitated by adding 80% (NH4)2SO4 followed by centrifugation (20,000xg, 30 min) at 4°C. Dissolved precipitate was dialyzed against 2 M (NH4)2SO4, 50 mM Tris-HCl, pH 7.8 and applied to a Hitrap Octyl FF (Cytiva) column equilibrated with 2 M (NH4)2SO4, 50 mM Tris-HCl, pH 7.8. Protein was eluted from the column with a gradient of decreasing concentration of (NH4)2SO4. Fractions were examined by Western blotting with anti-Cry j 2 monoclonal antibody (Torii pharmaceutical) to choose fractions including Cha o 2 for further purification. The fractions were pooled, dialyzed against 0.5 M NaCl, 30 mM Na2HPO4/NaH2PO4, pH 7.8 and applied to a HiTrap IMAC HP column (Cytiva). Protein was eluted from the column with a gradient of decreasing pH by using 0.5 M NaCl, 30 mM Acetate/Na3Acetate (pH 4.0). The fractions including Cha o 2 were pooled and dialyzed against 0.2 M NaCl, 30 mM Acetate/Na3Acetatem, pH 4.0. The dialyzed sample was applied to a HiTrap SP HP (Cytiva). Protein was eluted from the column with a gradient of increasing concentration of NaCl.

*Purification of Cha o 3 (n Cha o 3)*

Chamaecyparis obtusa pollen (Yamizo pollen study group) was extracted at 1:20 (w/v) in 40 mM NH4HCO3, pH 7.8, for 3 hours at 4°C with gentle stirring. Supernatant was obtained by centrifugation (20,000xg, 30 min, 4°C). Proteins were precipitated in the presence of 20% (NH4)2SO4. The supernatant was applied onto a Phenyl HP column (Cytiva). Proteins were eluted from the column with a gradient of decreasing concentration of (NH4)2SO4. Bands with apparent molecular weights of 50~75 Kd in SDS-PAGE were analyzed by nano-LC-MS/MS. Fractions including Cha o 3 were pooled, dialysed against 30 mM Acetate/Na3Acetate, pH 5.0 and applied to a HiTrap SP HP column (Cytiva). Protein was eluted from the column with a gradient of increasing concentration of NaCl. The fractions including Cha o 3 were selected by protein staining (Oriole, Bio-rad) of SDS-PAGE gel. Protein concentration was determined by the BCA method. Purified protein was further assessed by Western blot analysis with an anti-Cha o 3 rabbit antisera raised against Cha o 3 synthetic peptide (N-SVRSRWNYDASVKG-C) in our hands. We diluted the purified n Cha o 3 1000 times with PBS and used it for BAT.

**Sodium dodecyl sulfate polyacrylamide gel electrophoresis (SDS-PAGE) and Western blot analyses**

Purified allergens were mixed with Sample buffer (TEFCO, Tokyo, Japan) and then denatured at 95ºC for 5 minutes and separated by SDS-PAGE. Precision Plus ProteinTM unstained Standards (Bio-Rad, CA) were used as molecular weight standards. Gel images stained with SyproRuby were captured by ImageQuant™ LAS 4000 (Cytiva, Uppsala, Sweden).

Allergens separated using SDS-PAGE were electrically transferred to Immobilon®-PSQ PVDF Membrane (Merck KGaA, Darmstadt, Germany). The membranes were blocked with BlockAce (KAC, Kyoto, Japan) for 1 hour. The membranes were subsequently incubated with a primary antibody or antiserum for overnight at 4°C. After washing 5 times with PBS-T (0.05% Tween20) buffer, Mouse IgG HRP-conjugated Antibody (R&D systems, MN) or Rabbit IgG HRP-conjugated Antibody (R&D systems) were used as a secondary antibody and incubated for 1 hour at room temperature. After washing 5 times with PBS-T, ECL Prime Western Blotting Reagent (Cytiva) was used as a chemiluminescent substrate. Chemiluminescence signals were captured using the ImageQuant™ LAS 4000. Images were analyzed with ImageQuant TL (Cytiva) to estimate the purity of the protein.

**Basophil activation test**

A commercial kit (Allergenicity kit; Beckman Coulter) was used to quantify basophil CD203c expression according to the manufacturer’s instructions. Briefly, whole blood samples (100 μl) were incubated with PBS, anti-IgE antibody, Japanese cypress pollen crude extract (30 μg/ml), or various concentrations of purified n Cha o 1, n Cha o 2 (1, 3, 10 ng/ml) and n Cha o 3 (1, 10, 100 ng/ml) mixed with CRTH2-FITC/ CD203c-PE/ CD3-PC7 reagent for 15 minutes at 37°C. Then, the percentage of CD203chigh/CRTH+/CD3- cells was determined by BD Accuri™C6 flow cytometry (Becton Dickinson). All blood samples were tested for CD203c expression within 2 hours after collecting with EDTA as an anticoagulant. $\geq$5% increase of CD203c^+^ basophils compared with PBS upon the stimulations was considered to be positive.

**Measurement of allergen specific IgE titers**

Plasma samples were analyzed for Japanese cypress Cha o 1 and Cha o 2 specific IgE titers by ImmunoCAP (Phadia 200, Thermo Fischer Scientific) according to the manufacturer’s instructions. The inhibition assay was assessed by Japanese cypress ImmunoCAP in the presence of competing allergen in the liquid phase. 50 μL of plasma was mixed with 100 μL of PBS, JCy extract (10 μg Protein/mL), purified n Cha o 1 (10 μg/mL), purified n Cha o 2 (10 μg/mL) or purified n Cha o 3 (10 μg/mL) for 2 hours at 37°C prior to performing the JCy ImmunoCAP assay.

**Statistical analysis**

For correlation coefficient determination, statistical analyses were performed using the Pearson correlation coefficient. p< 0.05 was considered to be significant.

**References**

E1. Popescu FD, Vieru M. [Precision medicine allergy immunoassay methods for assessing immunoglobulin E sensitization to aeroallergen molecules.](https://pubmed.ncbi.nlm.nih.gov/30519536/)

World J Methodol. 2018;8:17-36

E2. Kimura Y, Kuroki M, Maeda M, Okano M, Yokoyama M, Kino K. Glycoform analysis of Japanese cypress pollen allergen, Cha o 1: a comparison of the glycoforms of cedar and cypress pollen allergens. Biosci Biotechnol Biochem. 2008;72(2):485-91.

E3. Mori T, Yokoyama M, Komiyama N, Okano M, Kino K. Purification, identification, and cDNA cloning of Cha o 2, the second major allergen of Japanese cypress pollen. Biochem Biophys Res Commun 1999;263(1):166-71.

E4. Yasueda H, Saito A, Sakaguchi M, Ide T, Saito S, Taniguchi Y, et al. Identification and characterization of a group 2 conifer pollen allergen from Chamaecyparis obtusa, a homologue of Cry j 2 from Cryptomeria japonica. Clin Exp Allergy 2000;30(4):546-50.

**Figure legends**

**Figure E1: The amino acid sequence of purified n Cha o 3 was verified by LC-MS/MS**

The sequence of coverage of Cha o 3 was shown. 39% of the deduced amino acid sequence of Cha o 3 (C0HLA0) was identified by LC-MS/MS analysis of the purified protein (n Cha o 3) in this work. Matched peptides were shown as the bold red character.

**Figure E2: SDS-PAGE and Western blotting of the purified n Cha o 1, 2 and 3.**

A. SDS-PAGE analysis (left panel) and Western blot analysis (right panel) of purified Cha o 1 (n Cha o 1). As reported, Cha o 1 was migrated as doublet in SDS-PAGE gel (left panel). ^E2^ Purified Cha o 1 was detected with anti-Cry j 1 monoclonal antibody (mAb) in a dose-dependent manner (right panel). In contrast, Cha o 2 and 3 were not detected with anti-Cry j 1 mAb even when 1000 ng of the purified Cha o 2 and 3 were loaded (right panel).

B. SDS-PAGE analysis (left panel) and Western blot analysis (right panel) of purified Cha o 2 (n Cha o 2). Purified Cha o 2 was detected with anti-Cry j 2 mAb in a dose-dependent manner (right panel). In contrast, Cha o 1 and 3 were not detected with anti-Cry j 2 mAb even when 1000 ng of the purified Cha o 1 and 3 were loaded (right panel).

C. SDS-PAGE analysis (left panel) and Western blot analysis (right panel) of purified Cha o 3 (n Cha o 3). Single band with molecular weight in SDS-PAGE of about 65 KD Cha o 3 was detected with anti-Cha o 3 peptide rabbit antiserum (right panel). The 45 or 50kDa band in the JCy extract lane of the SDS-PAGE (left panel) should be Cha o 1 protein, which were not detected in Western blot (right panel) by using the rabbit polyclonal serum against Cha o 3.

**Figure E3: Correlation of n Cha o 1 and 2-specific IgE with JCy-specific IgE**

**in 27 patients.**

The serum titers of n Cha o 1 (r^2^=0.9622, p<0.0001) and n Cha o 2 (r2=0.9439, p<0.0001) - specific IgE show positive correlation to JCy crude extract-specific IgE, respectively (Pearson correlation coefficient).

**Figure E4: Negative control for basophil activation test.**

A representative basophil activation stimulated with PBS, anti-IgE antibody, Japanese cypress pollen (JCy) crude extract, or 10 ng/ml of n Cha o 1 or n Cha o 2 or n Cha o 3 from one subject who did not have JCy specific IgE.

**Figure E5: n Cha o 3 did not affect n Cha o 1 and n Cha o 2-induced basophil activation in 3 patients with JCy pollinosis**

Basophil activation test stimulated with 10 ng/ml of n Cha o 1 and n Cha 2 with or without Cha o 3 in 3 patients with JCy pollinosis (n=3).
